# Editorial: Emerging roles of extracellular matrix in the physiology and pathophysiology of the central nervous system

**DOI:** 10.3389/fncel.2024.1400652

**Published:** 2024-04-04

**Authors:** Bhanu P. Tewari, Katherine Conant

**Affiliations:** ^1^Department of Neuroscience, University of Virginia School of Medicine, Charlottesville, VA, United States; ^2^Department of Neuroscience, Georgetown University Medical Center, Washington, DC, United States

**Keywords:** perineuronal nets (PNNs), plasticity, extracellular matrix (ECM), brain, pathophysiology, neuron, glia, matrix metalloproteinase

Brain extracellular matrix (ECM) is a heterogeneous meshwork of interconnected biomolecules that fill the extracellular space. Despite occupying ~20% of brain volume (Syková and Nicholson, 2008), the complexity of the ECM remained obscure for nearly a century. Improved microscopy and tissue labeling techniques have revolutionized the ECM field, revealing distinctive structures such as basement membranes, perineuronal nets (PNNs), and interstitial matrix as well as the dynamic nature of ECM turnover (Celio, 1999). Physiological spatiotemporal changes in the ECM regulate several processes in the developing and adult brain, including neuronal migration, axonal growth, synaptic maturation and plasticity, and ionic and neurotransmitter homeostasis (Sorg et al., 2016; Fawcett et al., 2019; Tewari et al., 2023). On the other hand, abnormal ECM and PNN remodeling has been associated with several CNS pathologies including glioma, addition, traumatic injury, epilepsy, neurodevelopmental, neurodegenerative, and neuropsychiatric diseases (Bradbury et al., 2002; Soleman et al., 2013; Sorg et al., 2016; Riga et al., 2017; Lorenzo Bozzelli et al., 2018; Tewari et al., 2018; Alaiyed et al., 2020; Carceller et al., 2022).

Our current understanding suggests there exists a wide range of structural and functional attributes of brain ECM and PNNs, however, the molecular mechanisms by which ECM and PNN facilitate these functions remain under investigation. Moreover, how diseases modify ECM and PNNs, and the extent to which ECM remodeling is causally associated with CNS pathology, require further study. In this Research Topic, we focused on the emerging roles of ECM and PNNs with a focus on mechanistic insight.

The spatiotemporal versatility of the PNNs and ECM components depends on the expression and functional activity of their master regulators, matrix metalloproteinase (MMPs), and their inhibitors (Lorenzo Bozzelli et al., 2018). MMP9 is one of the major MMPs expressed in the brain and governs ECM and PNN remodeling to facilitate neuroplasticity. Accordingly, the expression of MMP9 is tightly regulated at multiple levels (Tewari et al., 2022). One of the important yet underappreciated regulatory mechanisms of MMP9 is the single nucleotide polymorphism (SNP) at position−1562C/T within the MMP-9 gene. An updated account of MMP9 SNP and its impact on the overall MMP activity in the course of neuropsychiatric disorders has been reviewed by Pabian-Jewuła and Rylski. This review article provides a comprehensive and in-depth analytical insight into the involvement of this MMP9 SNP in multiple sclerosis, brain stroke, neurodegenerative disease, schizophrenia, brain tumors, and Guillain-Barre syndrome. With a balanced mix of evidence from clinical and basic research, this review put forward the idea of deeply examining the role of MMP9 polymorphisms in the development of brain diseases.

Studies in the last few decades have advanced our understanding of the regulatory roles of ECM in the learning and memory processes (Sorg et al., 2016). In the case of reward memory, ECM and PNNs regulated plasticity appears to be a double-edged sword as it influences brain circuitry involved in strengthening processing of natural rewards such as food, as well as the processing of maladaptive drugs of abuse. Valeri et al. summarize the role of ECM in both processes. The authors also draw attention toward current challenges that limit the idea of ECM-based pharmacotherapies followed by discussing the future directions that can potentially help to bridge this gap of knowledge. This article also provides a realistic picture of the magnitude of knowledge needed to catapult the ideas of ECM and PNN-oriented therapeutics from bench to bedside.

Aging is a major and progressively increasing risk factor for ischemic strokes (Yousufuddin and Young, 2019); however, a vast majority of mechanistic knowledge in the field comes from studies on young-age stroke models. It appears to be an important concern considering the plausibility that an aging brain may present with a unique ECM landscape that renders the brain highly susceptible to ischemic injury in the background of potentially impaired regeneration capacity. Chmelova et al. tackle this question by performing a transcriptomic and proteomic analysis of the brain tissues from young and aged mice followed by comparing it with the age-matched permanent middle cerebral artery occlusion (pMCAo) model of cerebral ischemia. The authors identified several genes and proteins with altered expression, suggesting their association with aging and ischemic stroke. Overall, this elegant study not only suggests a shift in the mechanisms associated with ECM formation and remodeling in aging and ischemic stroke, and also identifies potential targets to examine from a therapeutic perspective.

Collagen XIX is a non-fibrillary isoform of collagen expressed primarily in the mammalian cerebral cortex. Despite being sparsely expressed, collagen XIX is essential for PNN organization (Su et al., 2017). In the present study, Amos et al. provide evidence of collagen XIX's role, well beyond PNN assembly, in pheromone recognition and synapse formation in the olfactory bulb. The authors used several techniques including *in-situ* hybridization, immunohistochemistry, quantitative real-time PCR, and behavioral assays to assess the impact of a global, brain-wide, and cell-specific loss of collagen XIX using knockout mice. The authors report on regional and lamina-specific distribution of collagen XIX mRNA in a distinct subset of inhibitory neurons in the olfactory bulb and an impairment of pheromone recognition in collagen XIX null and brain-wide knockout mice. Mechanistically, their data suggest that a loss of collagen XIX disrupts excitatory synapse formation in the olfactory bulb leading to pheromone recognition deficits in mice. This milestone study not only reports a novel function of collagen XIX in pheromone recognition but also strengthens the idea of the brain region-specific function of ECM molecules.

Taken together, this Research Topic compiles original research and review articles that provide deep mechanistic insight and updates that enhance our understanding of ECM structure and function. Importantly, these studies also identify the important gaps and questions for future research in the field of brain ECM and PNN research.

## Author contributions

BT: Writing – original draft, Writing – review & editing. KC: Writing – review & editing.
